# Invasive *Streptococcus pneumoniae* in Children, Malawi, 2004–2006

**DOI:** 10.3201/eid1706.101404

**Published:** 2011-06

**Authors:** Jennifer E. Cornick, Dean B. Everett, Caroline Broughton, Brigitte B. Denis, Daniel L. Banda, Enitan D. Carrol, Christopher M. Parry

**Affiliations:** Author affiliations: Malawi-Liverpool-Wellcome Trust Clinical Research Programme, Blantyre, Malawi (J.E. Cornick, D.B. Everett, B.B. Denis, D.L. Banda, E.D. Carrol);; University of Liverpool, Liverpool, UK (J.E. Cornick, D.B. Everett, C. Broughton, E.D. Carrol, C.M. Parry);; University of Malawi College of Medicine, Blantyre (D.L. Banda);; Angkor Hospital for Children, Siem Reap, Cambodia (C.M. Parry)

**Keywords:** Streptococcus pneumoniae, pneumococcal disease, Africa, antibiotic resistance, serotype, children, Malawi, bacteria, dispatch

## Abstract

Of 176 invasive *Streptococcus pneumoniae* isolates from children in Malawi, common serotypes were 1 (23%), 6A/B (18%), 14 (6%), and 23F (6%). Coverage with the 7-valent pneumococcal conjugate vaccine (PCV) was 39%; PCV10 and PCV13 increased coverage to 66% and 88%, respectively. We found chloramphenicol resistance in 27% of isolates and penicillin nonsusceptibility in 10% (by using meningitis breakpoints); all were ceftriaxone susceptible.

*Streptococcus pneumoniae* causes a spectrum of disease, ranging from relatively mild otitis media to life-threatening pneumonia, meningitis, and septicemia. Recent estimates suggest that pneumococcal disease is responsible for 1 million deaths annually, >800,000 of which are in children <5 years of age in the developing world (*1*). Developing countries have the highest incidence of pneumococcal disease, and the spread of HIV, which increases the risk for pneumococcal disease up to 40-fold, has exacerbated the situation (*2*). In Malawi, in southern Africa, *S. pneumoniae* is 1 of the most common organisms isolated from blood and cerebrospinal fluid (CSF) cultures of children admitted to the hospital, and the case-fatality rate for invasive pneumococcal disease (IPD), pneumonia, septicemia, and meningitis is ≈25% (*3**,**4*).

The successful introduction of the 7-valent pneumococcal conjugate vaccine (PCV7) in several industrialized nations has led to plans to extend its use to sub-Saharan Africa (*2*). PCV7 contains the most commonly isolated 7 serotypes from IPD in children in the United States before vaccine implementation. However, these 7 serotypes account for <50% of IPD isolates from children in Africa (*5*). Surveillance of circulating serotypes is therefore essential information for developing policy about vaccine introduction.

In the United States, PCV7 has successfully reduced the incidence of IPD and antimicrobial drug resistance in vaccine serotypes; however, this decrease paralleled an increase in the incidence of IPD caused by nonvaccine serotypes, among which antimicrobial drug resistance is increasing (*6*). Resistance to penicillin and other antimicrobial agents in pneumococci complicates clinical management (*7*). Previous data from Malawi suggest that penicillin resistance in IPD is relatively low (*8**–**11*).

We report the serotypes of pneumococcal isolates from febrile children admitted to the largest hospital in Blantyre, Malawi, during April 2004–October 2006. We also report susceptibilities to antimicrobial drugs used to treat IPD.

## The Study

We studied *S. pneumoniae* isolated from the blood or CSF of children 2 months–16 years of age, admitted to Queen Elizabeth Central Hospital (QECH), the main referral hospital for southern Malawi, during April 2004–October 2006. Blood cultures were performed for all children admitted with signs of pneumonia or meningitis, and CSF cultures were performed for all children with signs suggestive of meningitis during the collection period. QECH admits ≈25,000 children and 17,000 adults annually and serves a population of ≈1 million. It is a government-funded teaching and referral hospital with 1,250 beds, although the total number of patients can exceed 2,000. Participants were recruited to studies of the host and bacterial factors determining outcome in invasive pneumococcal infection (*3*). The College of Medicine Research Committee, Malawi, and The Liverpool School of Tropical Medicine Local Research Ethics Committee granted ethics approval for this study.

Blood and CSF were processed by standard microbiological methods (*3*). *S. pneumoniae* isolates were identified by colony morphology and α-hemolysis and then confirmed by Gram staining and determination of optochin susceptibility (Oxoid, Basingstoke, UK). Isolates were stored at –80°C after primary isolation in bead and broth cryopreservers (Pro-Lab Diagnostics, Richmond Hill, ON, Canada). Isolates were transported to Liverpool and later subcultured for serotyping and MIC determinations.

Serotyping was performed by multiplex PCR as described by Pai et al. (*12*). MICs were determined by the Etest (AB Biodisk, Solna, Sweden) according to the manufacturer’s recommendation. Benzyl penicillin, ceftriaxone, and chloramphenicol were tested. *S. pneumoniae* ATCC 49619 was used as a quality control strain and gave values within an acceptable range. Antimicrobial drug susceptibility breakpoints were defined according to Clinical and Laboratory Standards Institute criteria (*13*).

We compared categorical values using Fisher exact test. A p value of <0.05 was considered significant. Statistical analysis was performed by using Stata 10 (StataCorp, College Station, TX, USA). When calculating serotype coverage, we assumed serotype 6A/B cross-protection for PCV7 and the 10-valent pneumococcal vaccine (PCV10).

A total of 180 isolates were collected from children admitted to QECH during the study period: 37 (21%) from CSF and 143 (79%) from blood. Four isolates did not remain viable during storage; 176 isolates were available for serotyping and MIC determination. Of these, 95 (54%) were from boys. Median age of patients was 2.5 years (range 2 months–14 years; interquartile range 8 months–7 years). Of the isolates studied, 100 (57%) were from HIV-positive children, and 71 (40%) were from HIV-negative children. Testing was declined for 5 (3%) children. The case-fatality rate was 25% for HIV-positive children and 21% for HIV-negative children (p = 0.68). Serotypes 1 and 6A/6B predominated, accounting for 23% and 18% of isolates, respectively (Table 1). Of the 176 isolates, 69 (39%), 116 (66%), and 154 (88%) had a serotype included in PCV7, PCV10, and PCV13, respectively.

**Table 1:** Serotypes of *Streptococcus.pnemoniae* isolated from children at Queen Elizabeth Central Hospital, Blantyre, Malawi, 2004–2006

Serotype	No. (%) isolates	HIV serostatus, no. (%)		
		Positive	Negative	Declined
1	41 (23)	16 (16)	24 (34)	1 (20)
6A/6B*	31 (18)	21 (21)	9 (13)	1 (20)
14*	11 (6)	8 (8)	3 (4)	0
23F*	11 (6)	7 (7)	4 (6)	0
12F	10 (6)	4 (4)	6 (8)	0
19F*	9 (5)	6 (6)	3 (4)	0
Sg18*	6 (3)	3 (3)	3 (4)	0
4*	5 (3)	5 (5)	0	0
7f	4 (2)	1 (1)	3 (4)	0
10A	3 (2)	2 (2)	1 (1)	0
16F	3 (2)	3 (3)	0	0
33F	3 (2)	1 (1)	2 (3)	0
35F	3 (2)	2 (2)	1 (1)	0
3	2 (1)	2 (2)	0	0
5	2 (1)	0	2 (3)	0
9V*	2 (1)	1 (1)	0	1 (20)
14/4	2 (1)	1 (1)	1 (1)	0
15a	2 (1)	1 (1)	1 (1)	0
19a	2 (1)	1 (1)	0	1 (20)
7c	1 (1)	0	1 (1)	0
8	1 (1)	0	1 (1)	0
34	1 (1)	1 (1)	0	0
11a	1 (1)	1 (1)	0	0
17f	1 (1)	1 (1)	0	0
35B	1 (1)	0	0	1 (20)
Not typeable	18 (10)	12 (12)	6 (8)	0
Total	176	100	71	5

*Serotypes in 7-valent pneumococcal conjugate vaccine: 4, 6B, 9V, 14, 18C, 19F, 23F. PCV10 adds serotypes 1, 5, and 7F. PCV13 adds serotypes 3, 6A, and 19A.

Clinical and Laboratory Standards Institute breakpoints for penicillin and ceftriaxone varied for meningitis or nonmeningitis infections (Table 2). Using the meningitis breakpoints, we found 158 (90%) isolates were susceptible to penicillin, and 18 (10%) were resistant to penicillin. However, according to non-meningitis breakpoints, all isolates were penicillin susceptible. Of the 150 children with meningitis, isolates from 16 (10.7%) patients were resistant by meningitis breakpoints. All isolates were susceptible to ceftriaxone by both breakpoints. Chloramphenicol resistance was present in 47 (27%) of isolates. Chloramphenicol-resistant pneumococci were isolated from 28 (39%) of the 71 HIV-negative children and 18 (18%) of the 100 HIV-positive children (p = 0.0027). The 2 groups did not differ significantly in levels of resistance to the other antimicrobial agents.

**Table 2:** Antimicrobial drug susceptibilities of *Streptococcus pneumoniae* isolates from children at Queen Elizabeth Central Hospital, Blantyre, Malawi, 2004–2006*

Antimicrobial drug	MIC, mg/L						No. (%) isolates*				Breakpoint values		
	Minimum	Maximum	MIC_50_	MIC_90_	GM		S	I	R		S	I	R
Benzyl penicillin													
Meningitis breakpoints	<0.016	0.500	<0.016	0.094	0.025		156 (90)	–	18 (10)		<0.06	–	>0.12
Nonmeningitis breakpoints	<0.016	0.500	<0.016	0.094	0.025		176 (100)	0	0		<2.0	4.0	>8.0
Ceftriaxone													
Meningitis breakpoints	<0.016	0.250	<0.016	0.064	0.025		176 (100)	0	0		<0.5	1.0	>2.0
Nonmeningitis breakpoints	<0.016	0.500	<0.016	0.094	0.025		176 (100)	0	0		<1.0	2.0	>4.0
Chloramphenicol	0.380	30.000	3.000	25.000	4.660		129 (73)	–	47 (27)		<4.0	–	>8.0

*MIC_50_, 50% MIC; MIC_90_, 90% MIC; GM, geometric mean; S, susceptible; I, intermediate; R, resistant; –, no intermediate resistance values for these antimicrobial drugs, according to Clinical and Laboratory Standards Institute definitions.

## Conclusions

Our study describes recent pneumococcal serotyping and antimicrobial drug susceptibility data for children in Malawi. Serotype distributions suggest that PCV7 would provide poor potential coverage for these children; PCV7 includes only 39% of serotypes identified. This information is supported by a previous study that found that PCV7 would cover 41% of invasive pneumococcal isolates from children (*14*). Serotypes 1 and 5, long regarded as essential in vaccines for use in sub-Saharan Africa, accounted for 23% and 1% of all isolates in this study, respectively. Our data suggest that use of the 13-valent vaccine, which includes serotypes 1 and 5 and is due to be introduced into Malawi in late 2011, will substantially increase vaccine coverage. The nontypeable isolates included in the study may have been typeable by an alternative method. However, the serotyping method used includes all serotypes in PCV7, PCV10, and PCV13.

Our study is not a formal epidemiologic study because it did not comprise a true random selection of isolates; however, QECH is the only public hospital in this area and most children admitted to the hospital live within the local community. Furthermore, we studied all consecutive cases during the study period, both severe and nonsevere. The sample of pneumococcal disease in children studied is therefore likely to be representative of the incidence of disease in this area.

The high proportion of blood cultures studied implies that pneumonia was the primary clinical diagnosis, however, most had meningitis. Penicillin is the first-line treatment for pneumonia and presumed sepsis at QECH, and ceftriaxone is the first-line treatment for suspected meningitis. Use of non-meningitis breakpoints in this study demonstrates no penicillin resistance. The susceptibility of all isolates to ceftriaxone confirms its suitability as a second-line treatment. Chloramphenicol resistance rates were high at 27%. The resistance levels reported here remain similar to those reported previously (*8**–**11*). Levels of resistance are comparable to those in other studies in sub-Saharan Africa but less than in many other areas in the world (*7*), possibly because antimicrobial drug use in Malawi is lower than that of other countries.
